# Affective vocalizations influence body ownership as measured in the rubber hand illusion

**DOI:** 10.1371/journal.pone.0186009

**Published:** 2017-10-05

**Authors:** Tahnée Engelen, Rebecca Watson, Francesco Pavani, Beatrice de Gelder

**Affiliations:** 1 Department of Cognitive Neuroscience, Faculty of Psychology and Neuroscience, Maastricht University, Maastricht, The Netherlands; 2 Department of Psychology and Cognitive Sciences, University of Trento, Trento, Italy; University of Bologna, ITALY

## Abstract

Emotional signals, like threatening sounds, automatically ready the perceiver to prepare an appropriate defense behavior. Conjecturing that this would manifest itself in extending the safety zone around the body we used the rubber hand illusion (RHI) to test this prediction. The RHI is a perceptual illusion in which body ownership is manipulated by synchronously stroking a rubber hand and real hand occluded from view. Many factors, both internal and external, have been shown to influence the strength of the illusion, yet the effect of emotion perception on body ownership remains unexplored. We predicted that listening to affective vocalizations would influence how strongly participants experience the RHI. In the first experiment four groups were tested that listened either to affective sounds (angry or happy vocalizations), non-vocal sounds or no sound while undergoing synchronous or asynchronous stroking of the real and rubber hand. In a second experiment three groups were tested comparing angry or neutral vocalizations and no sound condition. There was a significantly larger drift towards the rubber hand in the emotion versus the no emotion conditions. We interpret these results in the framework that the spatial increase in the RHI indicates that under threat the body has the capacity to extend its safety zone.

## Introduction

Emotional expressions are powerful triggers that at the same time communicate affective information, while also requiring situation-appropriate responses. Research has established the existence of a tight link between emotions and our bodies, and it is clear that perception of emotion stimuli in the environment prepare our bodies for action. This has been documented for the perception of emotional faces [1], bodies [2–4], and scenes [5,6], as well as sounds conveying affective information [7]. Especially when confronted with threatening information in our direct vicinity, we can expect physiological responses like a change in heart rate [8], skin conductance, pupil size [9], and pre-activation of our muscles [4]. This readiness for action is not only reflected in the body, but also in the brain, where it becomes apparent that motor cortex becomes more easily excitable [10], and areas involved in action preparation become active [3].

One important relation to explore is the one between action in response to emotions and body ownership. One way of testing changes in body ownership is with the so called Rubber Hand Illusion (RHI). The RHI presents a fascinating phenomenon in which synchronous visuo-tactile stimulation of a rubber hand and tactile stimulation of the real hand can lead to a person misplacing the location of their real hand. This misplacement is evident in a drift of the perceived location of the real hand towards the rubber hand, as well as in the subjective experience of the rubber hand being one’s own [11]. The illusion has been widely used to study the malleability of body ownership and reveals how, in this particular occurrence of multimodal integration, there is a strong dominance of the visual domain over touch and proprioception, possibly related to the fact that vision has far higher spatial acuity. Initial findings have since been extended and a multitude of factors have been found that seem to influence the strength of the illusion [12–15] and even transfer of the phenomenon to other body parts [16,17] or virtual hands [18].

Even though in their own right, the concepts of action preparation in response to emotions and conditions for ownership in the RHI have been widely studied, it has not been directly tested yet how emotions can influence our sense of body ownership. The current study sets out to probe the influence of affective vocalizations on the RHI. We used a between-subject design, in which the different groups were exposed to one of four sound conditions (happy vocal, angry vocal, non-vocal or no sounds) while experiencing the illusion. Each participant performed both asynchronous and synchronous stroking trials during the session, and both proprioceptive drift and a questionnaire on the subjective experience of ownership of the rubber hand were used to measure the strength of the illusion. To further disentangle effects between affective and neutral vocalizations, and to exclude the possibility that hearing voices per se can change the strength of the illusion, a second experiment was conducted. The set-up for the second experiment was similar as for the first; however, the groups were either presented with angry vocalizations, neutral vocalizations or no sounds as a control group. We hypothesized that emotional content of the vocalizations, and not neutral vocalizations or sounds that are of neutral content, will alter the strength of the illusion.

## Methods

### Participants

A group of 208 healthy Caucasian volunteers participated in this study (N = 114 in experiment 1(90 female, mean age (SD) = 22.5(3)), N = 94 in experiment 2(69 female, mean age (SD) = 23.4(5), minimum age for participation is 18). All participants were right handed and had normal or corrected to normal vision. Participants were unaware of the goal of the study until after the completion of the experiment. The study was performed in accordance to the Declaration of Helsinki and approved by the local ethical committee of Maastricht University. Written informed consent was obtained from all participants. Seven participants were excluded from the analysis because their average proprioceptive drift determined them as outliers in their group by deviating more than 2.5 standard deviations from the mean. For the analysis the following group sizes were included; 29 participants in the happy vocalizations group, 27 participants in the angry vocalization group, 27 in the no sounds group, and 28 in the non-vocal sounds group. For experiment 2 the distribution was as follows; 30 in the no sound group, 31 in the angry vocalizations group and 29 in the neutral vocalization group.

### Sound stimuli

Stimuli consisted of different sound categories; angry vocal, happy vocal, or non-vocal sounds in experiment 1 and additionally neutral vocal in experiment 2. Brief (< 2 seconds), affective (angry or happy) or neutral bursts (vocalizations) from females and males of different identities (5 males and 5 females), taken from the Montreal Affective Voices database [19], were compiled together to create separate sound clips each lasting 1.5 minutes long.

A condition of non-vocal sounds was included to ensure any observed effect of emotion was not induced by sounds in general and a no sound condition functioned as a baseline measure of the rubber hand illusion. This equivalent clip was made up of non(human)-vocal sounds (e.g., environmental, industrial, animal) taken from the voice localizer described in [19].

### Experimental set-up

**Fig 1:**
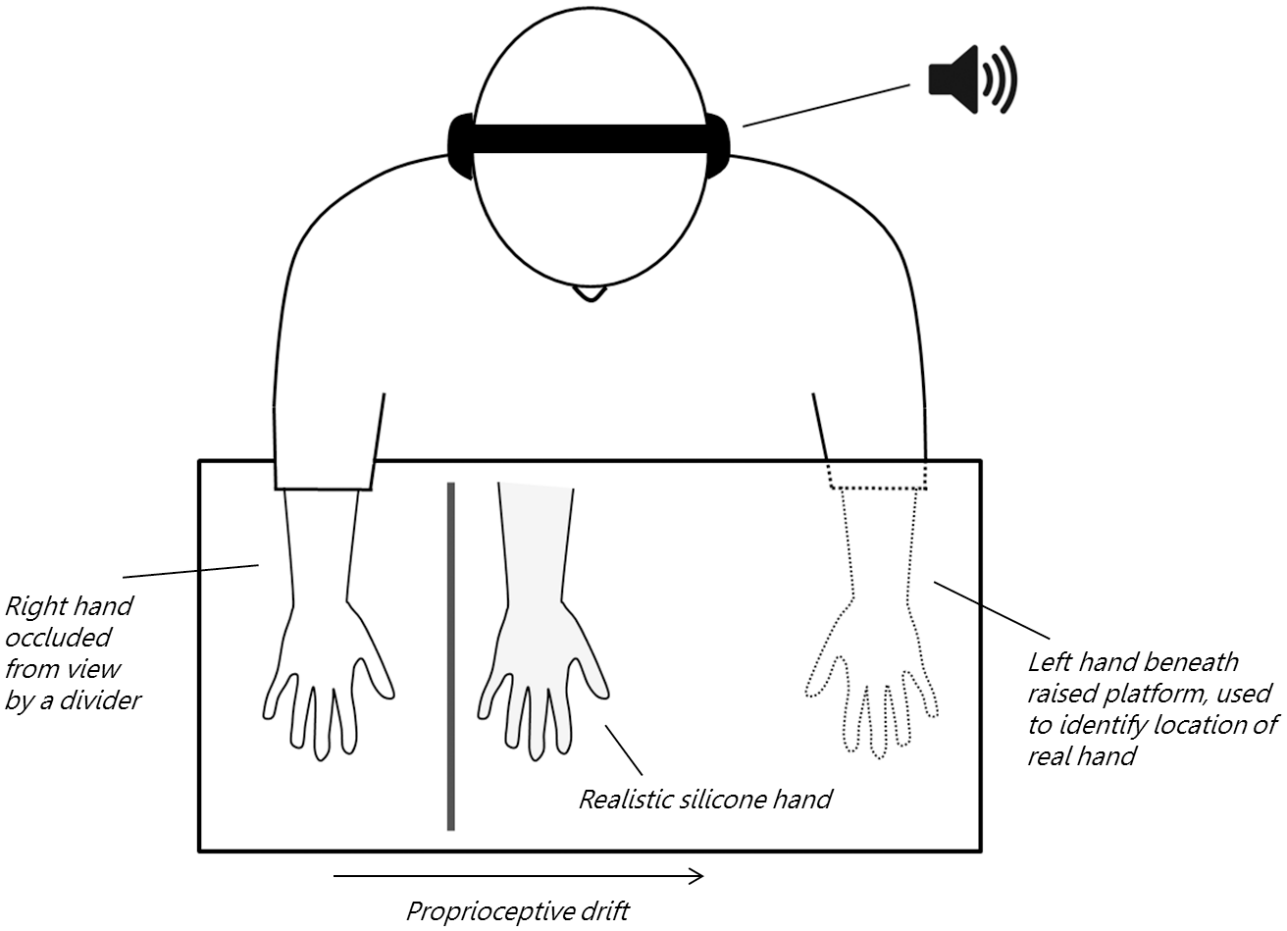
Experimental set-up. The participant is seated at a table on which a raised platform is placed. The participant’s right hand is placed on top of the platform, but occluded from view by a divider. The left hand is placed below the platform. During the experiment, participants focus their attention on a realistic silicone hand placed in front of them, while their real hand and the fake hand are brushed by the experimenter in either a synchronous or asynchronous fashion. At the end of each trial, participants use their left hand to indicate where they think their right index finger is located, by moving their left index finger over a tape measure placed on the platform on the side of the experimenter. Additionally, they answered nine questions related to their subjective experience during the trial.

Participants were seated behind a table on which a raised platform was positioned (measurements (LxWxH) 80x40x10cm). The participant was asked to place their right hand at the right side of the platform; ten cm to the left of their hand was a divider which blocked the view of their real hand. Ten cm to the left of the divider a realistic silicone hand was placed, on which they were asked to focus their attention during each trial. Before the start of the experiment participants were provided with over-ear headphones. In the angry vocal, happy vocal or non-vocal sound conditions, sounds were played through the headphones. In the no-sound condition, the headphones remained on the head but no auditory stimulation was delivered. Sound intensity was fixed to the same level for each participant by setting the intensity to a fixed volume on the laptop operated by the experimenter to play the sounds. Participants were asked to rest their left hand underneath the raised platform. After each trial participants were asked to close their eyes and use their left hand to indicate the perceived position of their real hand on a tape measure attached at the end of the platform facing the experimenter. Before this measure of proprioceptive drift was taken, the experimenter positioned the index finger of the participants’ left hand at the left end of the platform at varying positions, to rule out any learning effects. The position of the real hand was aligned with 0 cm on the tape measure, and the number of cm they deviated from 0 was noted by the experimenter. This was repeated twice after each trial, and the average of these two observations was used as the proprioceptive drift of the participant in that particular trial. During each trial the participant focused their attention on the rubber hand, while both their real hand and the rubber hand were stroked either synchronously or asynchronously by the experimenter. During the asynchronous trials the stroking of the real and the rubber hand was temporally incongruent. Each trial lasted 1.5 minutes, during the entirety of which a sound stimulus was played, if the participant was in one of the sound groups. See Fig 1 for a schematic overview of the experimental set-up.

To address some of the shortcomings in experiment 1 (see Discussion), a follow up experiment was conducted in which the procedure was slightly altered. To address any possible carry over effects, the real hand of the participant was revealed between each trial. Additionally, paint brushes were used to stroke the hands. To assure that any differences between conditions observed in experiment 1 were not only due to the sounds including voices or not, experiment 2 included a condition with neutral voices. Other than these differences the procedure was the same as in the first experiment.

### Design

Each participant performed six trials during the session, three asynchronous and three synchronous. After each trial the strength of the illusion was measured by assessing proprioceptive drift towards the rubber hand and a questionnaire as devised by [11] (see Table 1 for the nine questions). Participants were randomly assigned to one of the four conditions (angry, happy, non-vocal or no sound in experiment 1 or angry, neutral or no sound in experiment 2) and the order of questionnaire questions and synchronous or asynchronous trials was counterbalanced within participants.

**Table 1:** Questionnaire on subjective experience of the rubber hand illusion assessed after each trial.

Questionnaire
Q1. It seemed as if I were feeling the touch of the finger in the location where I saw the rubber hand touched
Q2. It seemed as though the touch I felt was caused by the finger touching the rubber hand
Q3. I felt as if the rubber hand were my hand
Q4. It felt as if my (real) hand were drifting towards the left (towards the rubber hand)
Q5. It seemed as if I might have more than one right hand or arm
Q6. It seemed as if the touch I was feeling came from somewhere between my own hand and the rubber hand
Q7. It felt as if my (real) hand were turning ‘rubbery’
Q8. It appeared (visually) as if the rubber hand were drifting towards the right (towards my hand)
Q9. The rubber hand began to resemble my own (real) hand, in terms of shape, skin tone, freckles or some other visual feature

### Analysis

For each participant the average proprioceptive drift was calculated separately for synchronous and asynchronous trials. Using this average drift as a dependent variable, a mixed ANOVA was run with the stroking condition (synchronous/asynchronous) as a within-subjects factor and sound condition (angry vocal/happy vocal/non-vocal/no-sound in experiment 1; no sound, angry vocal and neutral vocal in experiment 2) as a between-subjects factor.

As the data from the questionnaire were not normally distributed, WS and BS effects were separately assessed using non-parametric tests. To see how the BS factor (sound condition) influenced each question, the WS factor (stroking condition) was averaged and a Kruskal Wallis test was run for each question separately. Post-hoc tests included Mann-Whitney tests for each group compared to the no sound control, which a Bonferroni corrected *p*-value (*p*_bonf_ = 0.05/3 for experiment 1 and *p*_bonf_ = 0.05/2 for experiment 2).

To ensure there was a significant difference in the subjective experience of ownership between the synchronous and asynchronous stroking condition in general, we calculated the average score across questions for each stroking condition, and then compared the two using the Wilcoxon signed-rank test, ignoring the BS (sound condition) factor.

## Results

### The effect of emotion on proprioceptive drift towards the rubber hand

#### Experiment 1

**Fig 2:**
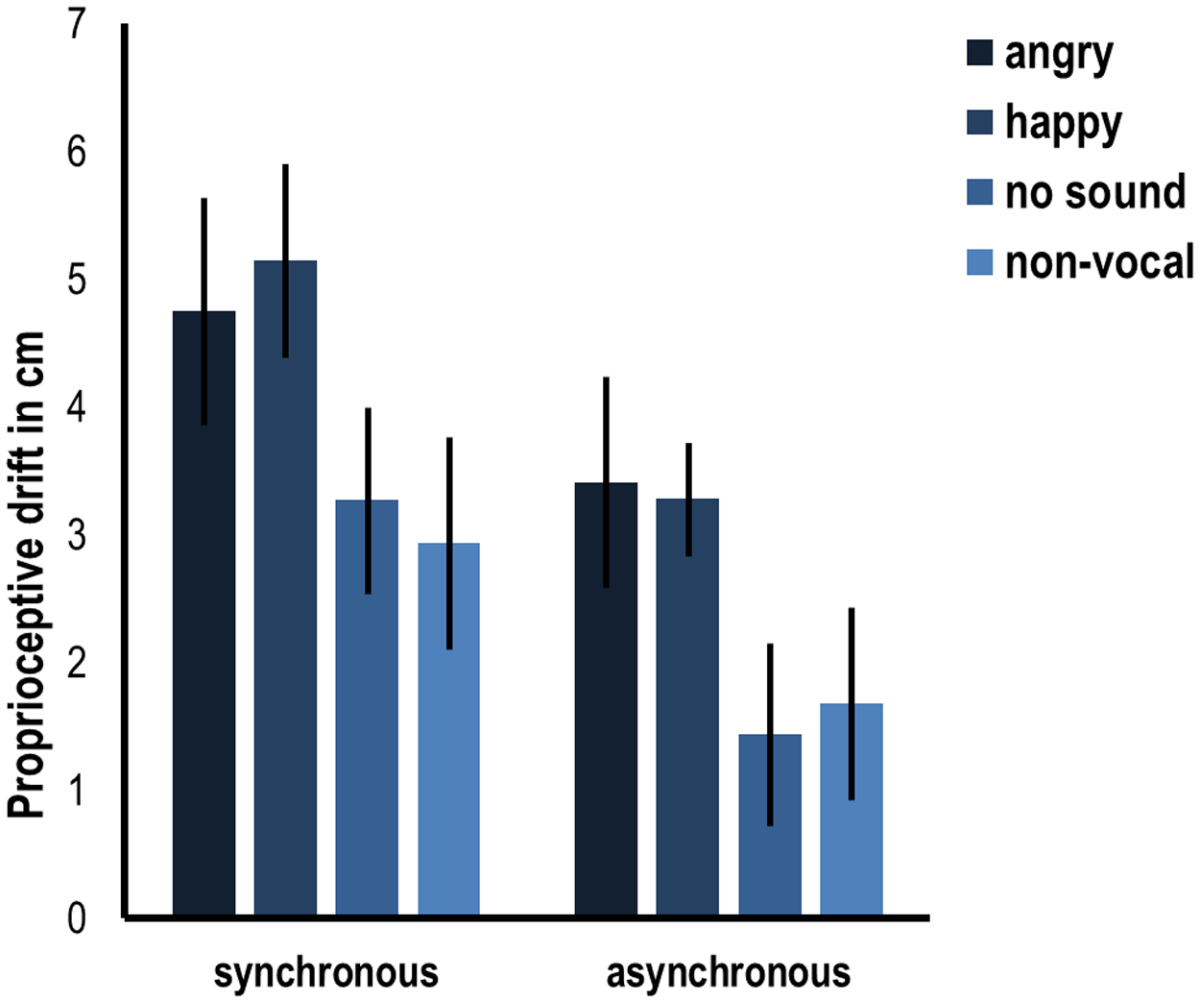
Average proprioceptive drift in cm in the different conditions of experiment 1 (angry vocal, happy vocal, no sound and non-vocal sounds) in synchronous or asynchronous stroking. Error bars indicate standard error.

The results of the mixed ANOVA on average proprioceptive drift showed a significant effect of stroking condition (*F*(1,107) = 35.483, *p* < .001), with higher proprioceptive drift in synchronous than in asynchronous stroking (respective means 4 and 2.5cm), confirming a successful induction of the rubber hand illusion when the rubber hand and the real hand are stroked synchronously. However, there was no interaction between stroking condition and sound condition (*F*(3,107) = .365, *p* = .779) and also no main effect of sound condition was evident in the data (*F*(3,107) = 2.209, *p* = .091). See Fig 2 for an overview of the results.

**Fig 3:**
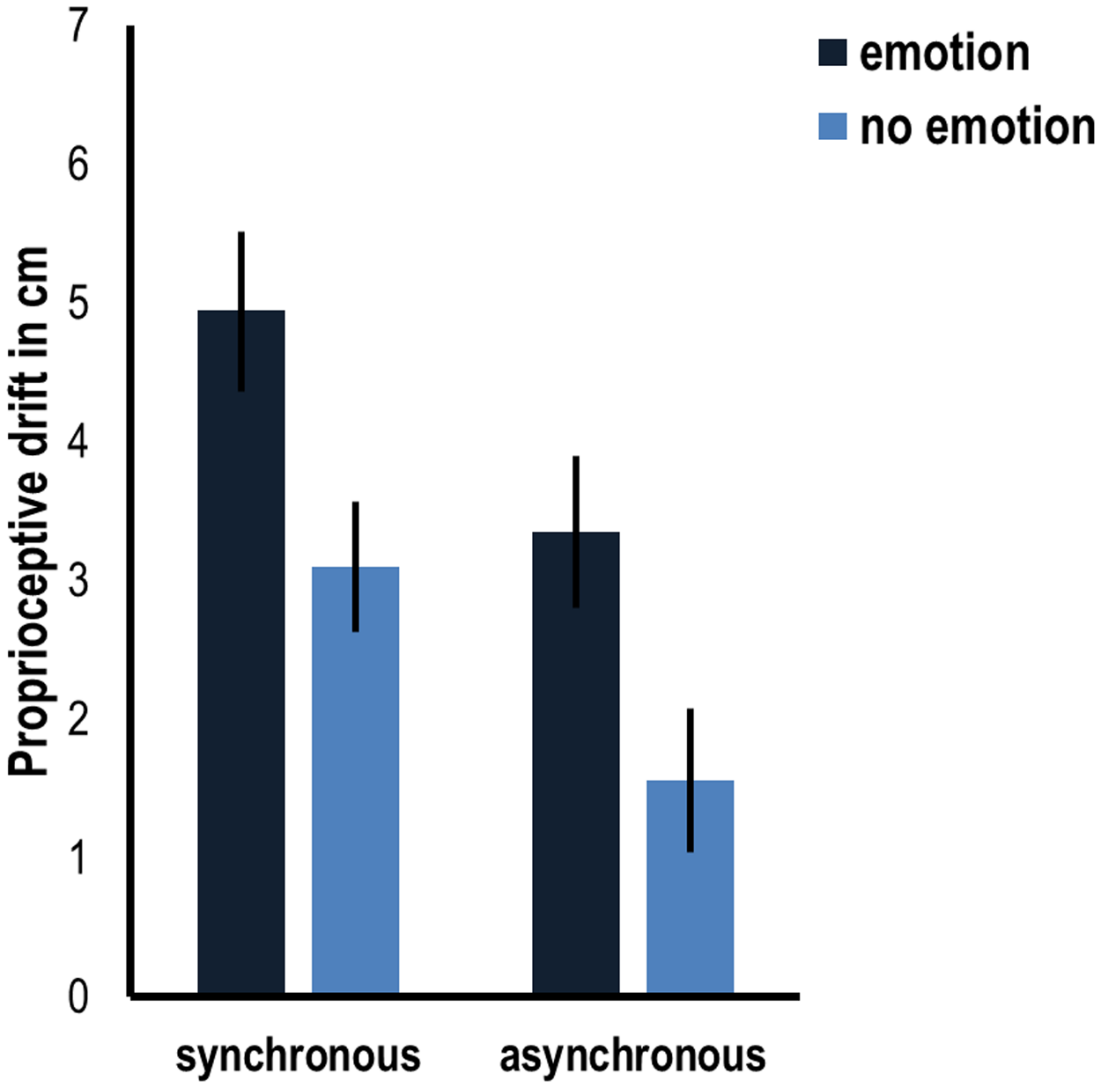
Average proprioceptive drift in cm for the data pooled into emotion (angry and happy vocal) and no emotion (no sound and non-vocal sounds) with synchronous or asynchronous stroking. Error bars indicate standard error.

Given the fact we had no *a priori* hypothesis that valence of the emotion would affect the illusion differently, we decided to pool the groups together into emotion (happy vocal and angry vocal) and no emotion (non-vocal and no sound). The results of this ANOVA revealed again a significant main effect of stroking condition ((*F*(1,109) = 35.480, *p* < .001), as well as a significant main effect of emotion (*F*(1,109) = 6.728, *p* = .011), which was driven by a higher proprioceptive drift in the emotion versus no emotion conditions (respective means are 4.1 and 2.3cm). There was no interaction between stroking condition and sound condition (F(1,109) = .01, *p* = .92). See Fig 3 for an overview of the results.

#### Experiment 2

**Fig 4:**
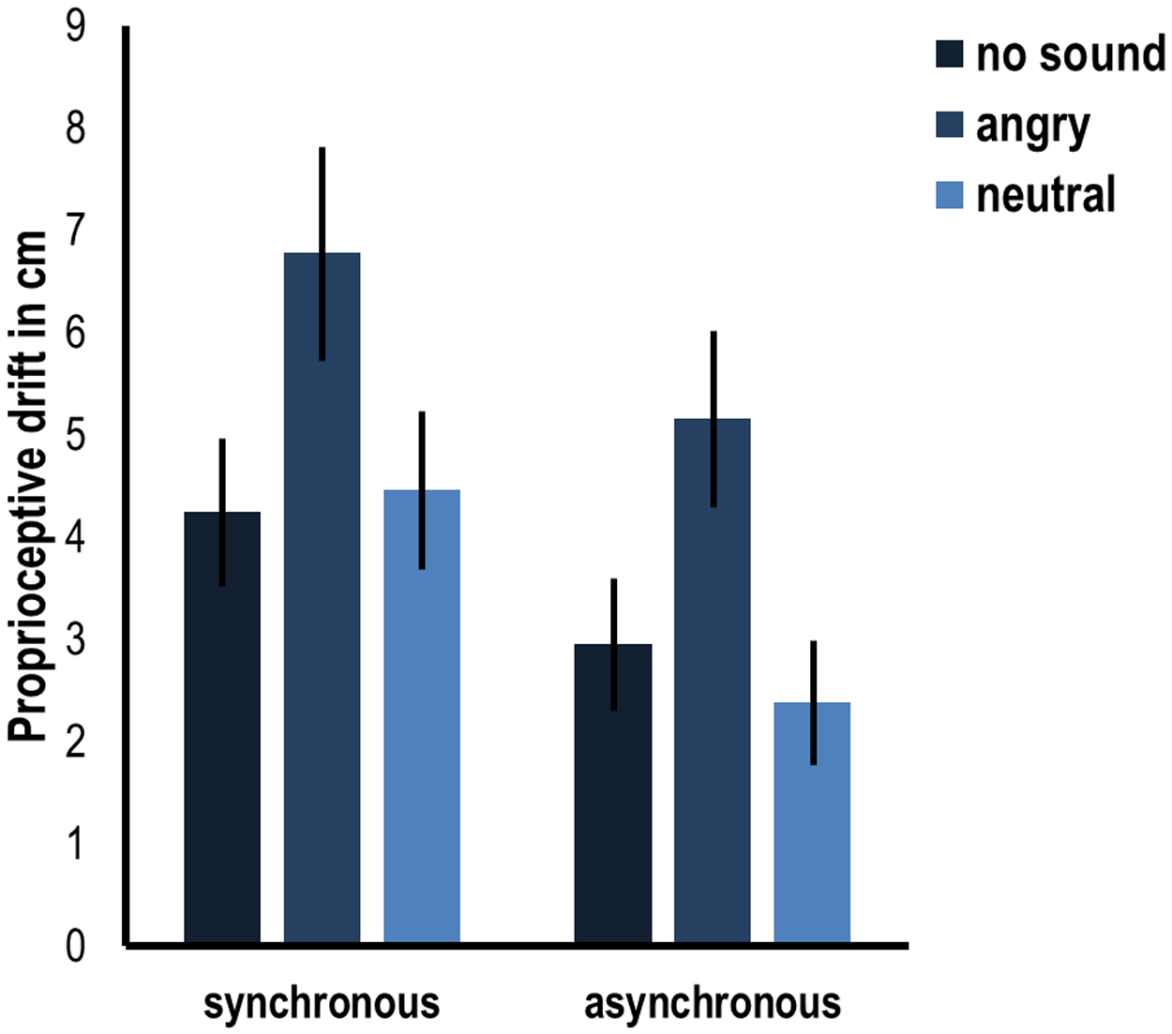
Average proprioceptive drift in cm in the different conditions of experiment 2 (no sound, angry vocal and neutral vocal) in synchronous and asynchronous stroking. Error bars indicate standard error.

The results of the mixed ANOVA on average proprioceptive drift measured in experiment 2 showed a significant effect of stroking condition (*F*(1,87) = 29.510, *p* < .001), as well as a significant main effect of sound condition (*F*(2,87) = 3.612, *p* = .031). Pairwise comparisons showed that this main effect was driven by trends towards significance in comparisons of no sound versus angry sounds (*p* = .083) and angry sounds versus neutral sounds (*p* = .057). No significant difference was apparent between the no sound and neutral sound condition (*p* = 1). There was no significant interaction between stroking condition and sound condition (*F*(2,87) = .537, *p* = .586). See Fig 4 for an overview of the results of experiment 2.

### The effect of emotion on the subjective experience of the rubber hand illusion as measured by a questionnaire

In experiment 1, no significant effects of sound condition were found on questions 1–8 (Q1: *H*(3) = 5.888, *p* = .117, Q2: *H*(3) = 1.351, *p* = .717, Q3: *H*(3) = 1.774, *p* = .621, Q4: *H*(3) = 2.371, *p* = .498, Q5: *H*(3) = 2.365, *p* = .500, Q6: *H*(3) = 5.643, *p* = .130, Q7: *H*(3) = 3.823, *p* = .281, Q8: *H*(3) = 2.593, *p* = .459), only question 9 showed a significant main effect (*H*(3) = 8.748, *p* = .033). Bonferroni corrected post-hoc tests for question 9 showed only a significant difference between the happy and no sound condition (*U* = 208.500, *p* = .007 < *p*_bonf_).

In experiment 2, there was no significant effect of sound condition reflected in any of the questions (Q1: *H*(2) = 0.714, *p* = .700, Q2: *H*(2) = 1.064, *p* = .588, Q3: *H*(2) = 0.218, *p* = .897, Q4: *H*(2) = 3.177, *p* = .204, Q5: *H*(2) = 0.690, *p* = .708, Q6: *H*(2) = 1.548, *p* = .461, Q7: *H*(2) = 1.404, *p* = .496, Q8: *H*(2) = 3.950, *p* = .139, Q9: *H*(2) = 2.214, *p* = .331).

**Fig 5:**
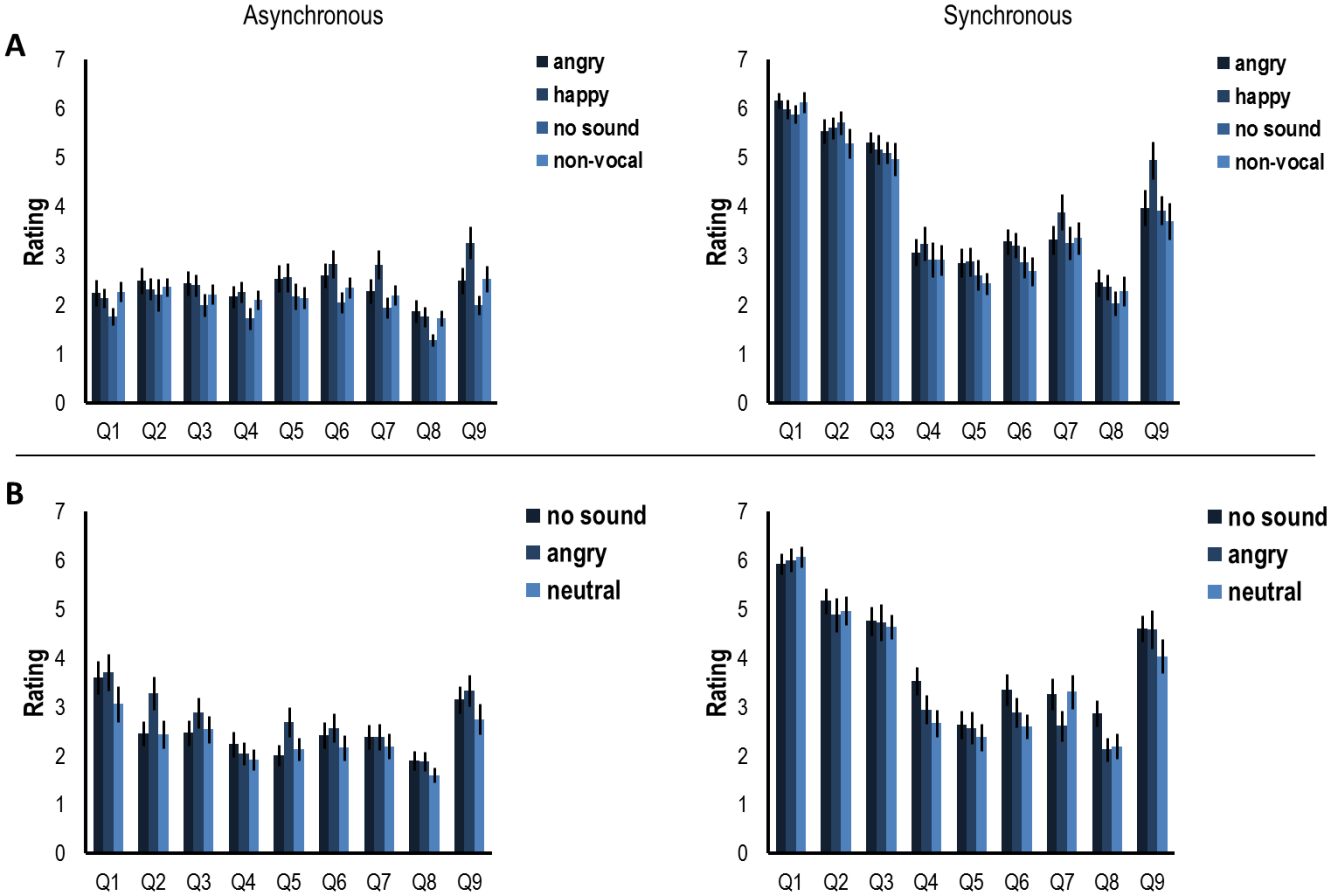
Average scores of the answers to the nine questions related to the subjective strength of the rubber hand illusion as measured in experiment 1 (A) and 2 (B). Error bars indicate standard error.

There was a main WS effect for stroking condition in both experiment 1 (*z* = -9.121, *p* < .001) and experiment 2 (*z* = -7.899, *p* < .001), showing that the subjective experience of ownership was higher for synchronous than asynchronous stroking. See Fig 5 for an overview of the questionnaire results for both experiments 1 and 2.

## Discussion

The current experiment set out to explore the influence of emotional stimuli on body ownership as measured in the rubber hand illusion. Specifically, we investigated if emotional vocalizations would alter the strength of the RHI as compared to a non-vocal and no sounds condition. Our main finding is that passively listening to emotional vocalizations caused a stronger illusion, as measured in the proprioceptive drift towards the rubber hand. This effect was again replicated in experiment 2, in which we found increased proprioceptive drift caused by angry vocalizations compared to both a no sound and neutral vocalization condition. This also illustrates that it is not the sound of voices per se causing the change in proprioceptive drift, rather the valence of the vocalization. No dissociation could be made between positive and negative vocal sounds. These results demonstrate for the first time that (auditory) emotion perception can influence body ownership and in turn our readiness to accept a rubber hand as our own.

The exact mechanism underlying the changes in body ownership in the RHI have been extensively debated, with accounts ranging from sole bottom-up processing [11,20], to an interoceptive predictive coding model, which puts interoceptive inference as a cornerstone of both experienced body ownership and emotion [21]. It is by now becoming clear that it is unlikely that the RHI is only driven by bottom-up mechanisms, and the roles of internal representations of the body and current bodily states seem more apparent [22,23]. For the illusion of ownership of the rubber hand to arise it is crucial that the rubber hand is in at least an anatomically plausible position, that the rubber hand is realistic, and stroking of the rubber hand and real hand is synchronous [24]. The illusion arises from a manipulation of the timing of multimodal exteroceptive feedback, and both interoceptive and other exteroceptive signals have been shown to influence the illusion.

It is interesting to start exploring body ownership in the light of its evolutionary purpose and what, and if, there actually is any meaning to the fact that in some instances we are more likely to accept something as being part of ourselves. In the ‘bodyguard hypothesis’[25] it is argued that one function of body ownership could be to play a motivational role for self-protection. The fact that response to threat to the rubber hand is correlated with the level of experienced ownership [26], already suggests involvement in self-defense and planning of protective movements. One of the sensorimotor representations of the body that we are able to experience is the protective body schema, and within this schema it is not necessarily only our biological body that we defend, but anything we experience as our body at that point in time. If a body part is incorporated into our body schema, we can experience it as our own, and the protective body schema presents a special case in which planning of defensive behavior is required. Anything that falls within our protective body schema is experienced as our own, and thus is worth defending. The results of the current experiment fall within this line of reasoning, as we observe an increase in proprioceptive drift when participants are presented with angry vocalizations. However, it is important to consider that we did not observe a difference between vocalizations of positive or negative valence. This seems to suggest that affective vocalizations have a general effect of expanding the boundaries of what we consider our body. Importantly, the methods used in the current experiment are not sensitive to subtle differences in the temporal dynamics of these effects. Whereas both positive and negative emotions motivate us to attend to our environment (see e.g. [27]), and affect the sensorimotor system in similar ways (e.g. [28,29]), a key difference might be timing. Whereas it might not be crucial to immediately respond when confronted with laughter, a scream demands our immediate attention. Given the set-up of the RHI paradigm, no conclusion can be drawn on any possible chronometric effects of emotion on body ownership. Future research is needed to dissect the observed effects; it could be of interest to include a measure of action readiness (e.g. muscle contraction as measured with EMG) to the RHI paradigm to specifically test the evolutionary purpose of body ownership.

One striking observation in the current study is that the effects of emotional vocalizations on proprioceptive drift were not only apparent in the synchronous, but also the asynchronous stroking condition. Typically, the asynchronous stroking condition is not supposed to induce the illusion of experiencing the rubber hand as your own, reflected in lower proprioceptive drifts and questionnaire scores compared to the synchronous stroking condition, both also evident in the current experiment. The second experiment tried to address the issue of possible trial-to-trial carry over effects by revealing the real hand of the participant after each trial. Despite this manipulation, there were still effects of sound condition apparent in both synchronous and asynchronous trials in experiment 2. One possible explanation for this could be that in the current study a hyper-realistic silicone hand was used, and many participants reported experiencing some degree of illusion already before the start of the experiment, by just having their own hand hidden from view and the rubber hand placed in an anatomically plausible position. Indeed, it has been shown that not only hand shape but also texture creates a stronger illusion [30], and that a vision only condition can create proprioceptive drifts as strongly as synchronous stroking [31]. If we are to assume the strength of the illusion was altered because being confronted with affective vocalizations placed the participants in a different state, in which for example a protective body schema is triggered, this might explain why the effects are seen in both synchronous and asynchronous trials. Critically though, main effects of stroking condition were always apparent, which shows that there was indeed a stronger illusion in synchronous than asynchronous trials.

The results of the current experiment show that perceiving emotional vocalizations have the potential to alter susceptibility to the RHI. Given that this illusion is a way of assessing malleability of body ownership, these results demonstrate that in certain contexts we might be more willing to integrate something as belonging to our body than in others. This study provides the first steps into seeing how emotions and body ownership might interact, but further research will be needed to examine the exact mechanism underlying their interaction.

## Supporting information

S1 Dataset (7Z)
